# Kinetics of cardiovascular and inflammatory biomarkers in paediatric dengue shock syndrome

**DOI:** 10.1093/oxfimm/iqae005

**Published:** 2024-06-03

**Authors:** Ho Quang Chanh, Huynh Trung Trieu, Hung Tran Kim, Vuong Huynh Ngoc Thien, Vu Ngo Thanh Huyen, Alexandra Moncada, Kieu Thanh Nguyen Thi, Huynh Thi Le Duyen, Ngan Nguyen-Lyle, Nguyen Lam Vuong, Phung Khanh Lam, Angela McBride, Tu Qui Phan, Tam Dong Thi Hoai, Bridget Wills, Sophie Yacoub

**Affiliations:** Oxford University Clinical Research Unit, Ho Chi Minh City, 72707, Viet Nam; Oxford University Clinical Research Unit, Ho Chi Minh City, 72707, Viet Nam; Hospital for Tropical Diseases, Ho Chi Minh City, Viet Nam; Hospital for Tropical Diseases, Ho Chi Minh City, Viet Nam; Hospital for Tropical Diseases, Ho Chi Minh City, Viet Nam; Oxford University Clinical Research Unit, Ho Chi Minh City, 72707, Viet Nam; St George's University of London, London, United Kingdom; Oxford University Clinical Research Unit, Ho Chi Minh City, 72707, Viet Nam; Oxford University Clinical Research Unit, Ho Chi Minh City, 72707, Viet Nam; Oxford University Clinical Research Unit, Ho Chi Minh City, 72707, Viet Nam; Oxford University Clinical Research Unit, Ho Chi Minh City, 72707, Viet Nam; University of Medicine and Pharmacy at Ho Chi Minh City, Ho Chi Minh City, Viet Nam; Oxford University Clinical Research Unit, Ho Chi Minh City, 72707, Viet Nam; Oxford University Clinical Research Unit, Ho Chi Minh City, 72707, Viet Nam; Centre for Tropical Medicine and Global Health, Oxford University, Oxford, United Kingdom; Hospital for Tropical Diseases, Ho Chi Minh City, Viet Nam; Oxford University Clinical Research Unit, Ho Chi Minh City, 72707, Viet Nam; Oxford University Clinical Research Unit, Ho Chi Minh City, 72707, Viet Nam; Centre for Tropical Medicine and Global Health, Oxford University, Oxford, United Kingdom; Oxford University Clinical Research Unit, Ho Chi Minh City, 72707, Viet Nam; Centre for Tropical Medicine and Global Health, Oxford University, Oxford, United Kingdom

**Keywords:** Dengue shock syndrome, Biomarkers, Vascular leakage, Glycocalyx, Inflammation, Natriuretic peptides, Severe dengue

## Abstract

Glycocalyx disruption and hyperinflammatory responses are implicated in the pathogenesis of dengue-associated vascular leak, however little is known about their association with clinical outcomes of patients with dengue shock syndrome (DSS). We investigated the association of vascular and inflammatory biomarkers with clinical outcomes and their correlations with clinical markers of vascular leakage. We performed a prospective cohort study in Viet Nam. Children ≥5 years of age with a clinical diagnosis of DSS were enrolled into this study. Blood samples were taken daily during ICU stay and 7–10 days after hospital discharge for measurements of plasma levels of Syndecan-1, Hyaluronan, Suppression of tumourigenicity 2 (ST-2), Ferritin, N-terminal pro Brain Natriuretic Peptide (NT-proBNP), and Atrial Natriuretic Peptide (ANP). The primary outcome was recurrent shock. Ninety DSS patients were enrolled. Recurrent shock occurred in 16 patients. All biomarkers, except NT-proBNP, were elevated at presentation with shock. There were no differences between compensated and decompensated DSS patients. Glycocalyx markers were positively correlated with inflammatory biomarkers, haematocrit, percentage haemoconcentration, and negatively correlated with stroke volume index. While Syndecan-1, Hyaluronan, Ferritin, and ST-2 improved with time, ANP continued to be raised at follow-up. Enrolment Syndecan-1 levels were observed to be associated with developing recurrent shock although the association did not reach the statistical significance at the *P* < 0.01 (OR = 1.82, 95% CI 1.07–3.35, *P* = 0.038). Cardiovascular and inflammatory biomarkers are elevated in DSS, correlate with clinical vascular leakage parameters and follow different kinetics over time. Syndecan-1 may have potential utility in risk stratifying DSS patients in ICU.

## Introduction

Dengue has emerged in the last two decades to be the most abundant vector-borne viral infection globally, with an estimated 100 million clinically apparent dengue infections each year. Factors such as international travel, urbanization, climate change, and trade globalization have driven this global expansion [1]. Dengue shock syndrome (DSS) is a potentially life-threatening complication which, although only occurring in a minority of cases, presents considerable clinical challenges in hyper-endemic settings. Currently, no antiviral agents or host-directed therapeutics are available to treat dengue and management relies on judicious fluid infusion and supportive care [2]. The lack of accurate and simple methods to quantify leakage remains a significant barrier to guide fluid replacement.

Vascular leakage, a hallmark of the disease, is a consequence of endothelial dysfunction and disruption of the endothelial glycocalyx layer (EGL), which regulates vascular permeability and tone [3, 4]. Recent pathogenesis studies in severe dengue showed that both binding of the virus and its non-structural 1 (NS1) antigen to endothelial cells (ECs), coupled with marked inflammatory and immune responses, can lead to endothelial dysfunction [3]. Evidence of reduced glycocalyx thickness together with increased plasma concentrations of EGL fragments, such as Syndecan-1, Heparan Sulfate, and Hyaluronan, in dengue patients suggests these products may be useful biomarkers and surrogates of leakage severity [5, 6].

Hyperinflammation is likely to play a key role in driving vascular leak in dengue. Ferritin is commonly used as a marker of inflammatory activity in many conditions [7]. Elevated levels are associated with worse clinical outcomes in many dengue studies [8–11]. Suppression of tumourigenicity 2 (ST-2), also known as the IL-33 receptor, has emerged as an important component in immune regulation and inflammation [12]. Elevated ST-2 levels have been associated with severe dengue, suggesting a potential link between ST-2 and disease severity [13–16]. Simultaneous investigation of inflammatory response together with vascular leak may shed light on the complex mechanisms underlying dengue pathogenesis.

Furthermore, cardiac natriuretic peptides (atrial and brain-type), markers of cardiac dysfunction, are associated with volume status and changes in volume status [17, 18]. Emerging evidence suggests they might also play a broader role in immune responses and vascular permeability [19]. Previous studies have demonstrated the relationship of atrial natriuretic peptide (ANP) with volume status and EGL shedding, which are also key phenomena in dengue shock syndrome [20–22]. Exploring the potential relationships of these natriuretic peptides to various markers and clinical features in this setting may provide novel insights into interactions between cardiovascular compensatory mechanisms, vascular leakage and immune responses in severe dengue.

We therefore conducted a prospective study to investigate these selected biomarkers in paediatric DSS: firstly investigating associations with clinical outcomes including recurrent shock, profound shock, and respiratory distress; secondly investigating the kinetics of these biomarkers over the disease course; and thirdly assessing correlations between these biomarkers and traditional methods of clinical vascular leakage assessment.

## Materials and methods

### Study design

We performed a prospective observational study at the Hospital for Tropical Diseases (HTD), Ho Chi Minh City, Viet Nam, between December 2018 and December 2020. Ethical approvals were obtained from the Oxford Tropical Research Ethics Committee and the HTD Ethics Committee. Written informed consent and assent, if applicable, were obtained from all parents/guardians and participants.

Children ≥ 5 years of age with a clinical diagnosis of dengue shock syndrome, who were admitted to the paediatric intensive care unit (PICU) and had not received fluid resuscitation elsewhere, were eligible for enrolment. Participants were reviewed at study enrolment and then daily until PICU discharge or for up to 4 days, and again at a follow-up (FU) visit 7–10 days after hospital discharge. Standardised clinical information was recorded, including findings of clinical examination and haemodynamic assessment. A full blood count was performed daily, whereas albumin, liver and renal function were tested at enrolment and then subsequently if clinically indicated. Serial point-of-care haematocrit measurements were obtained at predefined time-points, including at presentation to ICU with shock, and one, three and five hours after commencing fluid resuscitation. Percentage haemoconcentration was calculated as (enrolment—FU haematocrit/FU haematocrit) × 100, with an assumption that the haematocrit value measured at FU represents the normal value of the patient. Laboratory confirmation of dengue was carried out in batches using Dengue IgM Capture ELISA and Dengue IgG Capture ELISA kits (SD, Korea).

### Cardiovascular and inflammatory biomarkers

All patients had biomarker samples obtained at four time-points: at presentation with shock (day 0), with the routine morning bloods on ICU days 1 and 2, and at the FU visit. The following biomarkers were measured using commercial enzyme-linked immunosorbent assay (ELISA) kits—Syndecan-1 (Diaclone, France), Hyaluronan (R&D, United States), Ferritin (Arigo, Taiwan), NT-proBNP (Roche, Germany)—while LUMINEX kits were used to measure ST-2 and ANP levels (R&D, United States). The normal range of the biomarkers was defined based on published literature: 10–100 ng/ml for Hyaluronan, <20 ng/ml for Syndecan-1, <150 ng/ml for Ferritin, <50 ng/ml for ST2, <109 pg/ml for ANP, <125 pg/ml for NT-proBNP [23–28]. Summary information on the selected biomarkers is presented in Supplementary Table S1.

### Portable echocardiography

Point-of-care echocardiography was performed at the bedside at study enrolment and then daily by one of two investigators, using Samsung (2018–19) or Vivid IQ (GE) (2019–20) devices; the same investigator completed all scans for an individual patient. The echocardiograms included 2-dimensional, M-mode, and Doppler studies. Stroke volume index, cardiac index, and pleural effusion thickness were measured according to standardised techniques. The inter-device and inter-operator variability of the echocardiography were checked at regular intervals and were consistently <10%.

### Clinical management protocols

Patients were managed according to the Hospital for Tropical Diseases protocol for paediatric DSS. The guidelines specify a standard regimen of 35 ml/kg Ringer’s lactate solution over 3 h (15–10–10 ml/kg/h) for initial resusciation. Subsequently, all patients receive a standardised schedule of Ringer’s lactate, reducing the rate of fluid infusion at fixed time-intervals to 3 ml/kg/h over 7 h. A 3-h infusion of colloid solution (6% starch 130 or 200, depending on availability) is recommended for patients who present with decompensated shock or develop recurrent shock during fluid resuscitation (definitions below). The duration of intravenous fluid infusion in DSS management is usually around 24–48 h depending on the evolution of shock.

### Clinical definitions

Dengue shock syndrome was diagnosed clinically based on the 2009 WHO guidelines. Compensated DSS was diagnosed as pulse pressure narrowing to ≤20mmHg with signs of impaired perfusion, but with the systolic pressure maintained in the normal range for age. Decompensated DSS was defined by a pulse pressure of ≤10mmHg or hypotension for age or unmeasurable blood pressure.

Patients were confirmed dengue as having a positive IgM at enrolment or IgM seroconversion based on two consecutive specimens taken at least 2 days apart, with at least one specimen obtained during the convalescent phase. Patients were then classified into probable primary (i.e. the first) or probable secondary (i.e. a second or subsequent) infection based on IgG results. A probable primary infection was defined by a IgG seroconversion on paired samples. A propable secondary infection was defined by a positive dengue-specific IgG result on the first sample.

The primary clinical endpoint was ‘recurrent shock’, an indicator of ongoing plasma leak causing further cardiovascular compromise during the course of fluid resuscitation. Recurrent shock was defined as another episode of clinical shock (recurrent narrow pulse pressure, in association with tachycardia and cool extremities, and/or a rising haematocrit) occurring more than 6 h after achieving haemodynamic stability (normal BP, PP ≥25mmHg, and improved peripheral perfusion).

The secondary endpoints were respiratory distress and profound shock. Respiratory distress was defined as increased work of breathing or increased respiratory rate by age (age 3–5 years: >30 breaths/min, age 6–11 years: >25 breaths/min, and >12 years: >20 breaths/min) and/or requirement for respiratory support (nasal continuous positive airway pressure or mechanical ventilation). Profound shock was defined as either (i) requirement for two or more colloid boluses (including if given during initial shock resuscitation, and/or if given subsequently for management of recurrent shock or (ii) requirement for inotropes in addition to colloid therapy to maintain cardiovascular stability.

### Statistical analysis

The data are presented using median and interquartile range (IQR) for numeric variables and number of cases and percentage (%) for categorical parameters. Plasma levels of biomarkers were transformed to base-2 logarithm (log 2) before analysis, and then summarized by study time point: presentation with shock (day 0), day 1, day 2, and FU. We compared the log 2 of enrolment biomarker variables between patients with compensated and decompensated shock using the Wilcoxon rank sum test. Linear mixed effect models were used to assess temporal changes in biomarker levels during the ICU stay, with the log 2 of biomarker as the outcome of interest and the study timepoint as a categorical covariate. Logistic regression was employed to assess associations of log 2 of enrolment biomarkers with categorical clinical outcomes including recurrent shock, respiratory distress, and profound shock. All analyses were adjusted for day of illness at presentation with shock. Associations between plasma levels of biomarkers and other continuous parameters measured at enrolment were assessed using Spearman’s correlations. To informally adjust for multiplicity, a significance level of 0.01 was used for all comparisons. All analyses were performed using R version 4.2.1.

## Results

### Patient characteristics and outcomes

In total, 92 DSS patients were enrolled between December 2018 and December 2020. Two patients withdrew, leaving 90 patients with a complete dataset for the final analysis. All patients had laboratory-confirmed dengue, with 87 probable secondary infections and 3 probable primary infections.

The median age was 12 years (IQR 10–13) and 42% were female (Table 1). The median BMI was 20.1 (IQR 17.2–22.9). The median illness day at presentation with shock was 5 (IQR, 5–6). Most patients had a reduced stroke volume index (SVI) (median, 17.2 [IQR, 14.0–20.8] ml/m^2^) at presentation. The 17/90 (19%) DSS patients with decompensated shock had higher heart rates, a lower stroke volume index and higher urea at presentation than the group with compensated shock.

**Table 1.** Enrolment characteristics of patients with dengue shock syndrome (DSS) at ICU presentation with shock.

Characteristic	*n*	**Overall**, *N* = 90	**Compensated DSS**, *N* = 73	**Decompensated DSS^a^**, *N* = 17
Age, years	90	12 (10, 13)	12 (10, 13)	10 (8, 13)
Female sex	90	38 (42)	29 (40)	9 (53)
Day of illness at presentation, day	90	5 (5, 6)	5 (5, 6)	5 (5, 6)
BMI, kg/m^2^	90	20.1 (17.2, 22.9)	20.2 (17.4, 22.8)	19.2 (14.7, 22.9)
Heart rate, bpm	90	110 (105, 122)	110 (104, 120)	120 (110, 127)
Systolic BP, mmHg	84	100 (90, 100)	100 (90, 100)	100 (90, 102)
Diastolic BP, mmHg	84	80 (75, 86)	80 (70, 85)	90 (80, 95)
SVI, ml/m^2^	90	17.2 (14.0, 20.8)	17.5 (14.8, 21.0)	14.0 (11.3, 18.7)
CI, l/min/m^2^	90	1.8 (1.6, 2.2)	1.8 (1.6, 2.2)	1.6 (1.4, 2.0)
HCT, %	90	50 (48, 52)	50 (47, 52)	52 (50, 55)
Albumin, g/l	90	36.9 (33.1, 39.3)	36.8 (33.3, 39.3)	37.0 (33.0, 39.2)
Urea, mmol/l	33	3.7 (2.9, 4.6)	3.6 (2.9, 4.5)	5.7 (3.9, 9.1)

a Decompensated DSS is defined as pulse pressure ≤ 10 mmHg or hypotension for age or unmeasurable blood pressure.

Data are presented as absolute count (%) for categorical variables and median (IQR) for continuous data. N, number of patients; n, number of measurements; BMI, body mass index; BP, blood pressure; SVI, stroke volume index; CI, cardiac index; HCT, haematocrit; AST, aspartate aminotransferase; ALT, alanin aminotransferase.

Among the 16/90 (18%) patients who developed recurrent shock during the ICU admission, 75% experienced one episode while 25% had two episodes. The median times to recurrent shock were 14 and 25 h after commencing intravenous fluid, for the first and second episodes respectively. Profound shock occurred in 9/90 (10%) patients and 10/90 (11%) patients developed respiratory distress. There were no deaths in the study cohort and the median length of ICU stay was 3 (IQR 3–4) days. Individuals presenting with decompensated DSS were likely to have worse clinical outcomes compared to those with compensated shock (Table 2)—i.e. higher rates of recurrent shock and respiratory distress, and longer ICU stay.

**Table 2.** Clinical outcomes.

Characteristic	*n*	**Overall**, *N* = 90	**Compensated DSS**, *N* = 73	**Decompensated DSS^a^**, *N* = 17
Recurrent shock	90	16 (18)	10 (14)	6 (35)
1 Episode		12 (13)	7 (10)	5 (29)
2 Episodes		4 (4)	3 (4)	1 (6)
Respiratory distress	90	10 (11)	6 (8)	4 (24)
Profound shock	90	9 (10)	3 (4)	6 (35)
Percentage haemonconcentration^b^, %	90	32 (25, 42)	32 (23, 39)	48 (31, 52)
Total IV fluid volume, ml/kg	90	130 (122, 149)	129 (121, 144)	133 (125, 175)
Colloid, ml/kg	34	35 (35, 47)	35 (33, 43)	36 (35, 58)
Crystalloid, ml/kg	90	123 (117, 134)	125 (118, 136)	95 (89, 118)
IV fluid duration, h	90	27.3 (24.9, 30.9)	27.2 (24.3, 30.2)	27.4 (27.0, 35.3)
Time to 1st re-shock, h	16	14 (11, 17)	14 (8, 16)	16 (13, 17)
Time to 2nd re-shock, h	4	25 (25, 26)	25 (25, 25)	29 (29, 29)
PICU duration, day	90	3 (3, 4)	3 (3, 4)	4 (3, 4)
Death	90	0	0	0

a Decompensated DSS is defined as pulse pressure ≤ 10 mmHg or hypotension for age or unmeasurable blood pressure.

b Percentage haemonconcentration was calculated as (enrolment– FU haematocrit/FU haematocrit) × 100.

Data are presented as absolute count (%) for categorical variables and median (IQR) for continuous data. N, number of patients; n, number of measurements; IV, intravenous; PICU, paediatric intensivecare unit.

### Characteristics of cardiovascular and inflammatory biomarkers in DSS patients

#### At ICU presentation with shock

**Figure 1.**
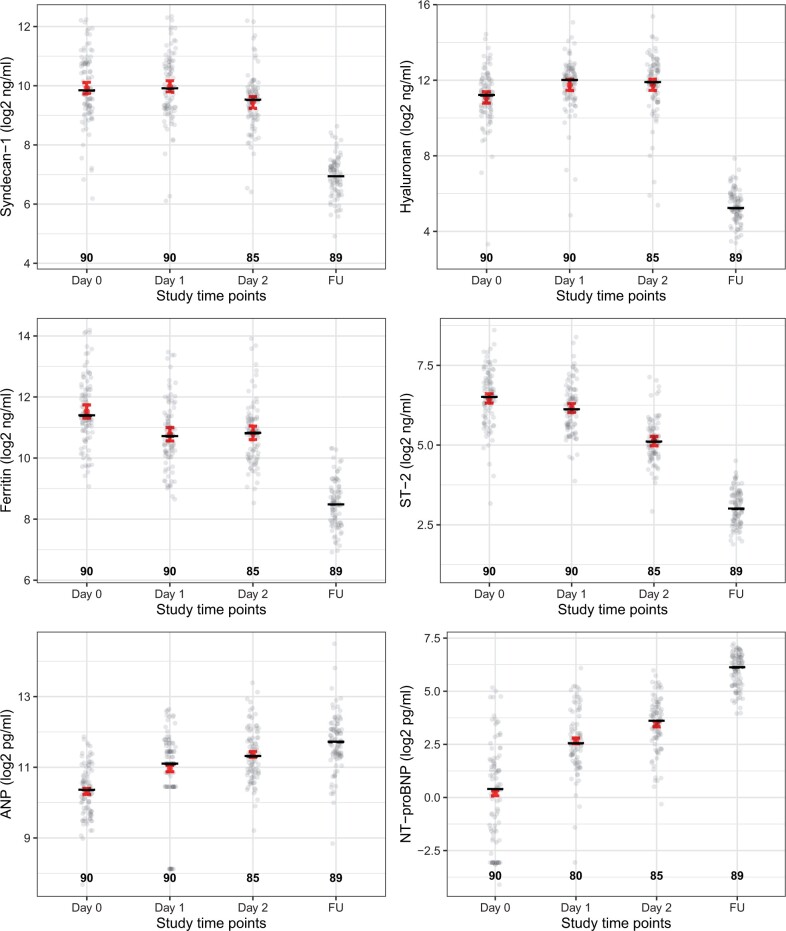
Serial measurements of cardiovascular and inflammatory biomarkers in patients with dengue shock patients during ICU admission and at the follow-up visit. All biomarker levels are transformed using log-2. The study timepoints were day 0—at presentation of shock, day 1, day 2, and at the FU (7–10 days after PICU discharge). Dots represent individual values in each group. The number represents the number of patients that contributed to each group. Short black line represents the median value of biomarkers during each study timepoint. The red point and error bars represent the predicted mean with its 95%CI calculated from the linear mixed effect models with the log 2 of plasma values of each biomarker (excluding values at follow up) as the outcome, the study timepoint as a categorical covariate, adjusted for day of illness at presentation with shock. ST-2, suppression of tumourigenicity 2; ANP, atrial natriuretic peptide; NT-proBNP: N-terminal pro brain natriuretic peptide

**Figure 2.**
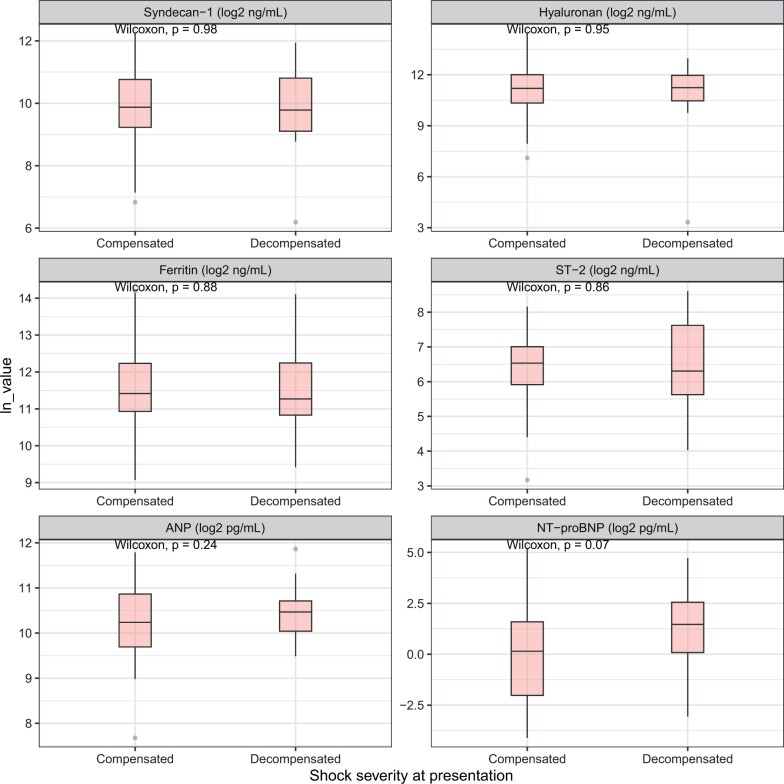
Boxplot of enrolment biomarkers, by shock severity. Decompensated DSS is defined as pulse pressure ≤ 10 mmHg or hypotension for age or unmeasurable blood pressure. All biomarker levels are transformed using log-2. Comparisons between group were assessed using Wilcoxon rank sum test. ST-2: suppression of tumourigenicity 2; ANP: atrial natriuretic peptide; NT-proBNP: N-terminal pro brain natriuretic peptide; p: *P*-value

Plasma levels of all biomarkers except NT-proBNP were elevated above the normal ranges at presentation with shock (Fig. 1, Supplementary Table S2) [19, 29–32], but no significant differences were observed between patients with compensated versus decompensated shock (Fig. 2).

**Figure 3.**
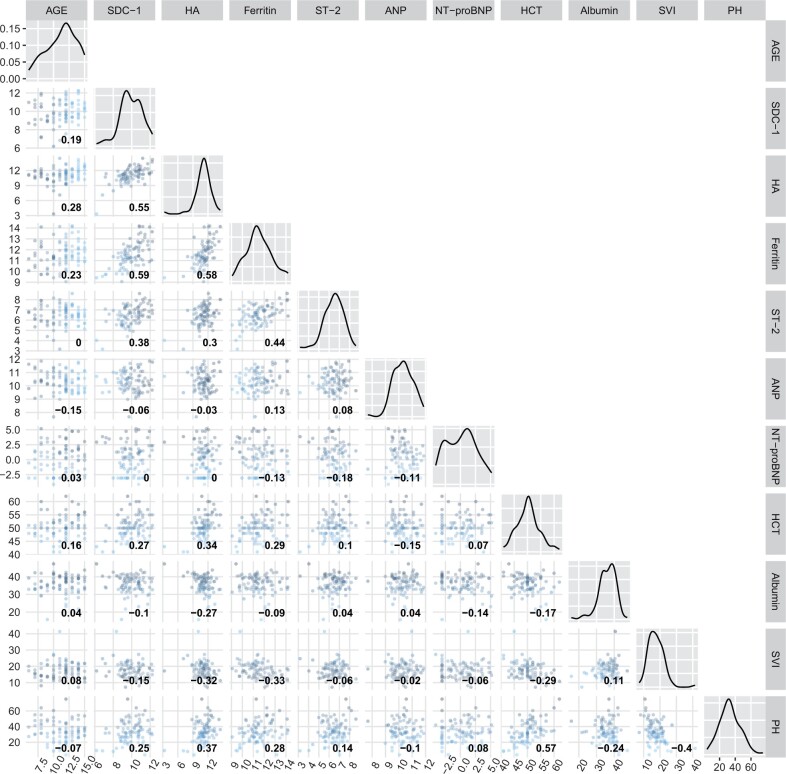
Pairwise correlation of biomarkers levels and markers of vascular leakage in DSS patients at presentation with shock. All biomarker levels are transformed using log-2. The red line is the linear regression line, and the gray region is the 95% confidence interval. The number inside each scatter plot represents the Spearman’s rank correlation coefficient of the two variables at the corresponding column and row. When the column and row refer to the same variable, the corresponding scatter plot is replaced by a density plot to reflect the distribution of that biomarker. Percentage haemoconcentration was calculated as (enrolment—FU haematocrit/FU haematocrit)*100. SDC-1: Syndecan-1, HA: Hyaluronan, ST-2: suppression of tumourigenicity 2; ANP: atrial natriuretic peptide; NT-proBNP: N-terminal pro brain natriuretic peptide, HCT: haematocrit, SVI: Stroke volume index, PH: Percentage haemoconcentration

We explored relationships between biomarker levels and other clinical markers of vascular leakage and volume status, measured at the same time point (presentation with shock). We found moderate positive correlations between Syndecan-1, Hyaluronan, Ferritin, and ST-2 levels (Fig. 3). Hyaluronan levels positively correlated with haematocrit (*r* = 0.34) and percentage haemoconcentration (*r* = 0.37), and negatively with stroke volume index (*r* = −0.32) and plasma albumin (*r* = −0.27). The correlations between Syndecan-1 and clinical leakage markers were similar but weaker compared to Hyaluronan (Fig. 3). Ferritin had a weak inverse relationship with Stroke volume index (*r* = −0.33). There was no correlation between any clinical leakage markers and ST-2, ANP, or NT-proBNP.

#### Dynamic changes over the course of disease

Plasma levels of the various biomarkers were presented at the four timepoints (Fig. 1). Measurement intervals after study enrolment (and thus initiation of fluid resuscitation) were fairly similar between individuals during the ICU stay—median 22 (IQR 18, 24) hours for the day 1 sample, and 45 (IQR 42, 47) hours for the day 2 sample (Supplementary Table S2).

**Figure 4.**
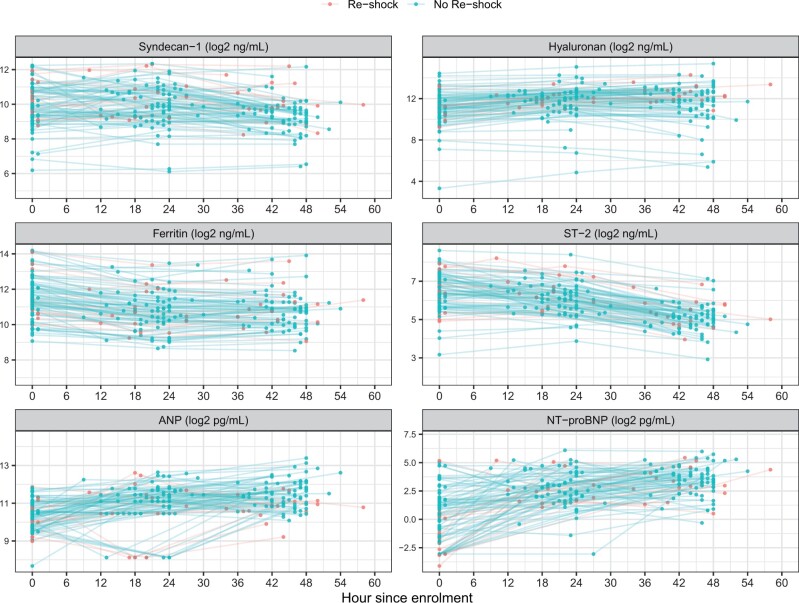
Kinetics of biomarker levels by individual during ICU admission, stratified by recurrent shock. All biomarker levels are transformed using log-2. Each coloured dot represents a biomarkers value. Coloured lines are changes of biomarker values for each patient. ST-2, suppression of tumourigenicity 2; ANP, atrial natriuretic peptide; NT-proBNP, N-terminal pro brain natriuretic peptide

The biomarkers followed different trajectories over the course of disease (Fig. 1, Supplementary Appendix SB). While Hyaluronan increased, Syndecan-1 remained unchanged over the first 24 hours (*P* = 0.549). Both biomarkers decreased by the day 2 timepoint. Ferritin, and ST-2 had already peaked at the time patients presented to ICU with shock and declined thereafter. These biomarkers were within the normal ranges at the follow-up visit. In contrast, the natriuretic peptides, including ANP and NT-proBNP, followed an increasing trend during ICU admission and continued to be raised at the follow-up visit. Of note, there is variability in the kinetics within individuals during the acute episode (Fig. 4).

### Utility of enrolment biomarkers in predicting recurrent shock and respiratory distress

At ICU admission with shock, patients with higher Syndecan-1 levels were more likely to develop recurrent shock (OR = 1.82, 95% CI 1.07–3.35, *P* = 0.038); however, after adjustment for multiple comparisons, the association did not reach statistical significance (at our predefined level of *P* < 0.01). There was also a trend for patients with lower NT-proBNP levels at ICU admission to develop recurrent shock (OR = 0.78, 95% CI 0.6–1.00, *P* = 0.071). There were no associations between enrolment biomarker levels and subsequent development of respiratory distress (Table 3). There were also no associations between any of the biomarkers and profound shock (Supplementary Table S3).

**Table 3.** Associations of enrolment biomarkers with recurrent shock and respiratory distress.

Enrolment value	Recurrent shock						Respiratory distress			
	No (*N* = 74)	Yes (*N* = 16)	OR	95% CI	*P*-value	No (*N* = 80)	Yes (*N* = 10)	OR	95% CI	*P*-value
**Hyaluronan (log2 ng/ml)**	11.17 (10.36, 11.99)	11.24 (10.42, 11.81)	1.32	0.78, 2.33	0.320	11.19 (10.35, 12.07)	11.24 (10.55, 11.60)	1.12	0.64, 2.04	0.706
**Syndecan-1 (log2 ng/ml)**	9.80 (9.15, 10.77)	10.21 (9.47, 10.88)	1.82	1.07, 3.35	0.038	9.85 (9.24, 10.76)	9.89 (9.14, 11.25)	1.35	0.76, 2.59	0.323
**Ferritin (log2 ng/ml)**	11.43 (10.90, 12.24)	11.20 (10.63, 12.25)	1.45	0.81, 2.74	0.223	11.40 (10.89, 12.23)	11.55 (10.62, 12.99)	1.66	0.86, 3.45	0.143
**ST-2 (log2 ng/ml)**	6.51 (5.87, 7.10)	6.62 (5.83, 7.17)	1.47	0.76, 3.04	0.266	6.53 (5.80, 7.09)	6.40 (6.29, 7.58)	1.71	0.79, 4.07	0.197
**ANP (log2 pg/ml)**	10.37 (9.77, 10.90)	10.02 (9.74, 10.66)	0.65	0.29, 1.45	0.297	10.32 (9.75, 10.75)	10.80 (10.01, 11.15)	1.84	0.73, 5.06	0.212
**NT-proBNP (log2 pg/ml)**	0.88 (-1.50, 1.94)	−1.43 (-3.06, 0.36)	0.78	0.60, 1.00	0.071	0.47 (-1.74, 1.86)	−0.27 (-1.71, 0.55)	0.95	0.71, 1.24	0.696

Data are presented as median (IQR). All biomarker levels are transformed using log-2. All analyses were based on logistic regression with recurrent shock or respiratory distress as the outcome, the log 2 of biomarker as the covariate, adjusted for illness day at presentation with shock. ST-2, suppression of tumourigenicity 2; ANP, atrial natriuretic peptide; NT-proBNP: N-terminal pro brain natriuretic peptide; OR: odds ratio; CI: confidence interval.

## Discussion

Overall, the results from this study have shown that several cardiovascular and inflammatory biomarkers are elevated at admission to ICU with DSS and correlate with clinical markers of vascular leakage. Although we found no difference in these biomarkers between patients presenting with compensated or decompensated shock, there was a trend toward higher levels of syndecan-1 in those who subsequently developed recurrent shock. While the glycocalyx and inflammatory biomarkers improved with time, the atrial and BNP natriuretic peptide remained high at the follow-up visit.

Our results add to the current literature on severe dengue by presenting dynamic changes of cardiovascular biomarkers coupled with detailed haemodynamic and clinical assessments. The selected biomarkers reflect the different yet related pathways of dengue-induced vascular leak syndrome. Shedding of the EGL, coupled with the hyperinflammatory response, increases vascular permeability, leading to slow but persistent plasma leakage during acute dengue [3]. However, once shock is established, evidence of the evolution of glycocalyx disturbance and hyperinflammation is limited, and it is still not clear whether tracking the end products of these pathways could aid clinicians in the prognostication or management of DSS. The pre-fluid resuscitation values of Syndecan-1, Hyaluronan, Ferritin, and ST-2 (measured at presentation with shock) in our study are higher than the values reported by other dengue studies [5, 10, 15, 16, 33–35], which is likely explained by more severe disease in our cohort. However, none of the biomarkers of glycocalyx degradation, inflammation, or cardiac dysfunction could differentiate patients by shock severity at ICU admission. One of the major components of the EGL, enrolment Syndecan-1, was observed to be higher in patients who developed recurrent shock although the differences did not reach the significance threshold of 0.01. This difference was however not seen with Hyaluronan, another critical component of the EGL. One reason for this discrepancy could be discordant glycocalyx component shedding. First, although several EGL components may be shed during severe dengue infection, the timing of release may vary. Early injury to the EGL layer may involve cleavage of the surface glycosaminoglycan Heparan Sulphate, which is bound to core proteoglycan Syndecan-1, and also to shedding of the loosely bound hyaluronan from its receptor CD44 [34]. Shedding of the Heparan Sulfate side chains may in turn render Syndecan-1 more vulnerable to subsequent proteolytic cleavage [5]. Thus by extension, shedding of Syndecan-1 may reflect more severe structural and functional damage to the EGL, potentially explaining the trend toward increased Syndecan-1 in patients with more severe vascular leakage [5, 33]. Second, Hyaluronan is unstable in a non-sulphated form and is more likely to be influenced by non-specific changes in shear stress during hypovolaemia and also to interstitial washout secondary to fluid loading [36, 37]. Indeed, the additional shear stress induced by fluid loading may contribute to the shedding of Hyaluronan, which might explain rising Hyaluronan levels over the first 24 h of fluid resuscitation [38]. Our finding may highlight while perservation of the glycocalyx layer may help protect the vasculature from extensive glycocalyx damage, therapeutics may need to be targeted to syndecan-1 and its axis.

With respect to biomarkers of inflammation, the trajectories of Ferritin and ST-2 suggest that inflammation may peak at or before presentation with shock. Although Ferritin and ST-2 have been associated with development of severe dengue in several cohorts [9, 15, 16, 35], in this study we found no relationships between Ferritin, ST-2 and our chosen outcomes; in this context, it may be that these biomarkers are closely linked with development of shock, but do not differentiate outcome once shock is established. In addition, the downward trend of inflammatory biomarkers after enrolment may reflect the fact that all the DSS patients in this study recovered without developing a macrophage activated syndrome or secondary haemophagocytic lymphohistiocytosis [8]. These results emphasise that trials of host directed immunomodulation for dengue may need to carefully select patients based both on the timing of presentation to hospital and the severity of inflammation, in order to identify the subset of patients most likely to benefit from such therapeutic interventions.

To our knowledge, this is the first study to systematically report values of ANP and NT-proBNP in paediatric DSS. Interestingly, ANP values, usually considered markers of cardiac stretching, were elevated at shock onset while NT-proBNP levels, usually increased in association with heart failure, were low. Regarding the ANP levels, our results differ from previous observations indicating that ANP is released in response to hypervolemia and atrial distention which occur during fluid resuscitation [20]. Putensen *et al.* however described an increase in ANP in hypovolaemic haemorrhagic shock; the authors hypothesized that ANP may be increased due to a combination of endothelin released after endothelial damage and an increase in circulating catecholamines causing vasoconstriction and subsequently increased cardio-pulmonary pressure [39]. This explanation may be relevant to our study, since increased systematic vascular resistance, evidenced by a gradual raise in diastolic blood pressure, is one of main compensatory mechanisms in response to hypovolemia in dengue. Yet we observed no significant right heart dilatation to suggest high cardiopulmonary pressure (data not shown). Another potential explanation is that the renal receptors for ANP could be downregulated leading to reduced ANP clearance, a phenomenon which has been seen in rats with severe haemorrhage [40]. In contrast, the NT-proBNP levels were markedly lower than the normal range at all the timepoints in our study, indicating there was no significant cardiac failure in our patient cohort. However, there was discordance between ST-2 and NT-proBNP levels, two FDA-approved markers of heart failure, which may reflect the fact that ST-2 can be released due to inflammatory responses as well as myocardial stress. Finally, both natriuretic peptides are small molecules (3 and 8.5 kD for ANP and NT-proBNP respectively), so they may have leaked out of hyperpermeable microvessels. The upward trajectories observed during admission and after discharge were unexpected, but may be due to the end of vascular leakage during the acute phase and the extended reabsorption of extravascular fluid during late convalescence resulting in cardiac stretching. Further research is warranted to confirm this finding.

A thorough search of relevant literature yielded our study is the first to provide a detailed overview of the kinetics of biomarkers representing different mechanisms of plasma leakage in dengue shock patients. However, the study only recruited paediatric patients, which may limit the generalisability of our findings, as it is becoming increasingly evident that adults and children exhibit important differences in dengue pathogenesis [35]. Further studies in adults are required to investigate whether there are similarities or important differences in the mechanisms underlying the leakage.

Secondly, the sample size was small and adverse outcome events occurred infrequently. This likely hindered our ability to detect true differences between subgroups, especially given the wide distribution of values we observed for some of the biomarkers. Furthermore, recurrent shock usually occurs within the first 24 h after admission with DSS, and some of the biomarkers we investigated have very short half-lives. Further studies to investigate prognostic biomarkers should consider focusing on the first 24 h and incorporating shorter measurement intervals in a larger cohort to validate our findings and to able to capture the kinetics more fully.

Finally, interpretation of such data is complex and ideally should take into account several dynamic factors—including changes in the production and/or cleavage of particular biomarkers over time, the possibility of differential leakage of biomarkers from the microvasculature, and the volume of distribution (i.e. the plasma volume) at the time of measurement. We selected recurrent shock as our primary endpoint to represent severe ongoing plasma leakage. Although this endpoint has real clinical relevance, its occurrence is very likely to be affected by the fluid therapy protocol used, as well as intrinsic physiological factors that regulate a particular individual’s compensatory response to hypovolaemia. Incorporating biomarker results into a robust predictive score that combines different measures of vascular leakage, haemodynamic status, compensatory reserve, inflammation, organ dysfunction, etc may prove to be a promising approach to translating our results into an effective tool for use in clinical practice.

## Conclusion

In conclusion, we have demonstrated the kinetics of biomarkers of EGL shedding and hyperinflammation in paediatric patients with established dengue shock during ICU admission. Enrolment Syndecan-1 may differentiate patients at higher risk of recurrent shock, potentially facilitating decision-making for clinical prioritization and management. This biomarker may serve as a criterion to select patients for personalised fluid therapy in future fluid resuscitation as well as host-targeted therapeutics trials.

## Supplementary Material

iqae005_Supplementary_Data

## Data Availability

The data underlying this article will be shared on reasonable request to the corresponding author.
