# Supplementary material for: Kinetics of cardiovascular and inflammatory biomarkers in paediatric dengue shock syndrome
Source: Oxf Open Immunol. 2024 Jun 3;5(1):iqae005. doi: 10.1093/oxfimm/iqae005 (PMC11211616; doi:10.1093/oxfimm/iqae005)
Supplement: iqae005_Supplementary_Data [file iqae005_supplementary_data.docx]

**Appendix A**

**Table S1. Summary of cardiovascular and inflammatory biomarkers investigated**

| **Biomarker** | **Description** | **Half life/ Molecular weight** | **Normal range** | **Summary of literature** | **Current findings in dengue literature** | **Values in dengue studies** |
| --- | --- | --- | --- | --- | --- | --- |
| **Hyaluronan** | - Glycoaminoglycan of endothelial glycocalyx (EG) - Non-sulfated GAG 🡪 more chemical vulnerable to EG degradation - Maintain vascular integrity and permeability, vasodilation | - 3-5 mins - Varying MW:70 - >1000kD | 10 - 100 ng/ml | - Increase in several conditions: inflammatory rheumatic diseases, infection (sepsis/septic shock), and cancer, etc. | - Increase in dengue (Tang et al., 2017), and different between severity (Surwato et al., 2017; Honsawek et al., 2007). - A predictor of DWS and correlated with high plasma NS1 levels (Lin et al., 2019); | - DF: 272 – 1580 ng/ml - DHF: 1324 – 4000 ng/ml - SD: 4700 – 7316 ng/ml |
| **Syndecan 1** | - Proteoglycan of EG - Maintain vascular integrity and permeability. | - 27 hours - 32 kD | < 20 ng/ml | - Increase in several conditions: sepsis and SOFA score in sepsis patients, cardiovascular disease, chronic kidney disease, diabetes, etc. | - Increase in dengue and correlated with plasma leakage grade. (Buijsers et al., 2021; Garishah et al., 2023; Lam et al., 2020; Mariappan et al., 2021; Suwarto et al., 2017) | - DF: 67 – 72 ng/ml - DHF: 56 – 95 ng/ml - Plasma leakage grade: 0: 112ng/ml; 1: 255 ng/ml; 2: 2613 ng/ml |
| **Ferritin** | - An iron storage protein in a non-toxic form - Secreted from activated macrophages and monocytes in response to pro-inflammatory cytokines, and released from damaged hepatic cells during infection | - 30 hours - 434 kD | < 150 ng/ml | - Not clear function in severe infection - One of the key diagnostic biomarkers of hyperinflammatory syndrome (Kotsaki et al., 2022; van der Poll et al., 2021) | A diagnostic and prognostic biomarker for dengue severity (Giang et al., 2018; Moras et al., 2022; Valero et al., 2019; van de Weg, Huits, et al., 2014);  Predictor of mortality in adults with dengue and sHLH (Kan et al., 2020) | - DF: 400 ug/l - DWS: 950 ug/l; - SD: 1100 ug/l |
| **ST2** | - Recepter of IL-33, a member of the IL-1 - Expressed on various immunological cells, and cardiac fibroblasts and cardiomyocytes - Secreted in response to cell damage, and myocardial stress - Participate in inflammatory T-cell mediated processes | - 5 hours - 37 kD | ≤50.0 ng/mL | - A strong, independent predictor of severity and mortality in cardiac failure | - A prognostic factor for severe dengue with the cut-off of 30 ng/ml (Teo A, et al., 2022) - Associated with disease severity and mortality (Houghto-Trivino et al, 2010; Guerrero CD et al, 2013; Arias J et al, 2014; Hsieh CC et al. 2019) | - DF: 17.8 ng/ml - DWS: 21.84 ng/ml - SD: 43.79 ng/ml |
| **ANP** | - Mainly synthesized by cardiomyocytes in the atria - Secreted in response to atrial wall stretching resulting from increased intravascular volume - Diuretic, natriuretic, and hypotensive activity, increased capillary permeability; moderate vasodilation of arteries and veins | - 2 mins - 3 kD | < 109 pg/ml | - A potential diagnostic marker in paediatric heart failure: identify children with heart failure, classify heart failure severity, discriminate cardiac load in paediatrics, and differentiate of underlying etiology (Gangus & Burckhardt, 2019) | No previous study | No previous data |
| **NT-proBNP** | - Mainly synthesized by cardiomyocytes in the ventricles - Secreted as a prohormone in a pulsatile pattern under volume and pressure stress - Diuretic, natriuretic, and hypotensive activity. | - 60-120 mins - 8.5 kD | < 125 pg/ml | - A diagnostic and prognostic biomarker of cardiac failure. | 12/81 (15%) adults with dengue had elevated Trop T or NT-proBNP | Dengue with elevated Troponin I and NT-proBNP: 1917 ng/ml |

| **Characteristic** | *n* | **Day 0**, N = 90 | *n* | **Day 1**, N = 90 | n | **Day 2**, N = 90 | *n* | **FU,** N = 90^1^ |
| --- | --- | --- | --- | --- | --- | --- | --- | --- |
| **Measurement interval***  **(hours)** | 90 | 0  (0, 0) | 90 | 22  (18, 24) | 85 | 45  (42, 47) | 89 | 220  (193, 240) |
| **Hyaluronan**  **(ng/mL)** | 90 | 2,382  (1,318, 4,076) | 90 | 4,128  (2,287, 5,842) | 85 | 3,826  (2,108, 7,046) | 89 | 38  (27, 56) |
| **Syndecan-1**  **(ng/mL)** | 90 | 921  (597, 1,746) | 90 | 965  (604, 1,779) | 85 | 740  (489, 934) | 89 | 123  (90, 159) |
| **Ferritin**  **(ng/mL)** | 90 | 2,701  (1,851, 4,840) | 90 | 1,685  (1,134, 2,705) | 85 | 1,811  (1,061, 2,501) | 89 | 358  (235, 485) |
| **ST-2**  **(ng/mL)** | 90 | 91  (58, 137) | 90 | 70  (49, 102) | 85 | 35  (26, 46) | 89 | 8  (6, 11) |
| **ANP**  **(pg/mL)** | 90 | 1,319  (866, 1,850) | 90 | 2,198  (1,401, 3,291) | 85 | 2,553  (1,840, 3,639) | 89 | 3,382  (2,572, 4,320) |
| **NT-proBNP**  **(pg/mL)** | 90 | 1  (0, 3) | 80 | 6  (3, 15) | 85 | 12  (7, 21) | 89 | 70  (39, 98) |
| **The measurement interval is the difference in hours between study enrolment and the time when the relevant blood sample was obtained. Study time points are presented as day 0 (i.e. presentation with shock), day 1, day 2 and follow-up (FU) (7 – 10 days after hospital discharge). Data are presented as median (IQR). N: number of patients, n: number of measurements.*  *ST-2: suppression of tumorigenicity 2; ANP: atrial natriuretic peptide; NT-proBNP: N-terminal pro brain natriuretic peptide.* | | | | | | | | |

**Table S2. Summary of plasma levels of cardiovascular and inflammatory biomarkers during ICU admission and at the follow-up**

**Table S3. Associations of enrolment cardiovascular biomarkers with profound shock in DSS patients**

| **Enrolment value** | **Profound shock** | | | | |
| --- | --- | --- | --- | --- | --- |
|  | **No (N = 81)** | **Yes (N = 9)** | **OR** | **95%CI** | **p-value** |
| **Hyaluronan**  **(log2 ng/ml)** | 11.25  (10.39, 12.00) | 11.19  (10.29, 11.28) | 1.07 | 0.57, 2.16 | 0.837 |
| **Syndecan-1**  **(log2 ng/ml)** | 9.87  (9.25, 10.76) | 9.78  (9.10, 11.41) | 1.52 | 0.82, 3.11 | 0.208 |
| **Ferritin**  **(log2 ng/ml)** | 11.43  (10.93, 12.23) | 10.83  (10.36, 12.77) | 1.38 | 0.65, 3.10 | 0.223 |
| **ST-2**  **(log2 ng/ml)** | 6.52  (5.87, 7.09) | 6.37  (5.34, 7.61) | 1.35 | 0.61, 3.28 | 0.473 |
| **ANP**  **(log2 pg/ml)** | 10.37  (9.74, 10.82) | 10.04  (10.00, 11.16) | 1.35 | 0.50, 3.88 | 0.297 |
| **NT-proBNP**  **(log2 pg/ml)** | 0.50  (-1.91, 1.86) | 0.08  (-1.69, 0.36) | 0.88 | 0.65, 1.12 | 0.479 |
| *Data was presented as median (IQR).*  *All biomarker levels are transformed using log-2.*  *All analyses were based on logistic regression with profound shock as the outcome, log2-transformed biomarkers as the covariate, and adjusted for illness day at presentation with shock. ST-2, suppression of tumorigenicity 2; ANP, atrial natriuretic peptide; NT-proBNP: N-terminal pro brain natriuretic peptide; OR: odds ratio; CI: confidence interval.* | | | | | |

**Appendix B - Results from linear mixed effect models for changes of biomarker levels during ICU admission**

1. **Hyaluronan**

|  | **Hyaluronan (log2 ng/ml)** | | |
| --- | --- | --- | --- |
| *Predictors* | *Estimates* | *CI* | *p* |
| Intercept | 9.72 | 8.71 – 10.72 | **<0.001** |
| Day 1 | 0.66 | 0.48 – 0.84 | **<0.001** |
| Day 2 | 0.68 | 0.50 – 0.86 | **<0.001** |
| DOI 5 at shock | 1.51 | 0.41 – 2.61 | **0.008** |
| DOI 6 at shock | 1.58 | 0.46 – 2.70 | **0.006** |
| DOI 7 at shock | 1.21 | -0.33 – 2.75 | 0.123 |
| N _USUBJID_ | 90 | | |
| Observations | 265 | | |

1. **Syndecan-1**

|  | **Syndecan-1 (log2 ng/ml)** | | |
| --- | --- | --- | --- |
| *Predictors* | *Estimates* | *CI* | *p* |
| Intercept | 9.32 | 8.61 – 10.03 | **<0.001** |
| Day 1 | 0.06 | -0.13 – 0.24 | 0.549 |
| Day 2 | -0.49 | -0.68 – -0.30 | **<0.001** |
| DOI 5 at shock | 0.53 | -0.24 – 1.30 | 0.176 |
| DOI 6 at shock | 0.81 | 0.02 – 1.59 | **0.045** |
| DOI 7 at shock | 0.81 | -0.27 – 1.89 | 0.141 |
| N _USUBJID_ | 90 | | |
| Observations | 265 | | |

1. **Ferritin**

|  | **Ferritin (log2 ng/ml)** | | |
| --- | --- | --- | --- |
| *Predictors* | *Estimates* | *CI* | *p* |
| Intercept | 10.58 | 9.87 – 11.30 | **<0.001** |
| Day 1 | -0.75 | -0.86 – -0.65 | **<0.001** |
| Day 2 | -0.67 | -0.78 – -0.57 | **<0.001** |
| DOI 5 at shock | 0.85 | 0.07 – 1.64 | **0.034** |
| DOI 6 at shock | 1.19 | 0.39 – 1.99 | **0.004** |
| DOI 7 at shock | 1.50 | 0.40 – 2.60 | **0.008** |
| N _USUBJID_ | 90 | | |
| Observations | 265 | | |

1. **ST-2**

|  | **ST-2 (log2 ng/ml)** | | |
| --- | --- | --- | --- |
| *Predictors* | *Estimates* | *CI* | *p* |
| Intercept | 6.13 | 5.61 – 6.65 | **<0.001** |
| Day 1 | -0.30 | -0.42 – -0.18 | **<0.001** |
| Day 2 | -1.37 | -1.50 – -1.25 | **<0.001** |
| DOI 5 at shock | 0.31 | -0.25 – 0.88 | 0.274 |
| DOI 6 at shock | 0.54 | -0.04 – 1.12 | 0.067 |
| DOI 7 at shock | -0.09 | -0.88 – 0.71 | 0.831 |
| N _USUBJID_ | 90 | | |
| Observations | 265 | | |

1. **ANP**

|  | **ANP (log2 pg/ml)** | | |
| --- | --- | --- | --- |
| *Predictors* | *Estimates* | *CI* | *p* |
| Intercept | 10.37 | 9.89 – 10.84 | **<0.001** |
| Day 1 | 0.64 | 0.42 – 0.87 | **<0.001** |
| Day 2 | 1.06 | 0.83 – 1.29 | **<0.001** |
| DOI 5 at shock | -0.21 | -0.72 – 0.29 | 0.401 |
| DOI 6 at shock | 0.14 | -0.38 – 0.65 | 0.593 |
| DOI 7 at shock | 0.05 | -0.66 – 0.75 | 0.894 |
| N _USUBJID_ | 90 | | |
| Observations | 265 | | |

1. **NT-proBNP**

|  | **NT-proBNP (log2 pg/ml)** | | |
| --- | --- | --- | --- |
| *Predictors* | *Estimates* | *CI* | *p* |
| Intercept | -0.75 | -1.67 – 0.17 | 0.110 |
| Day 1 | 2.47 | 1.97 – 2.98 | **<0.001** |
| Day 2 | 3.27 | 2.77 – 3.76 | **<0.001** |
| DOI 5 at shock | 0.95 | -0.01 – 1.92 | 0.053 |
| DOI 6 at shock | 1.13 | 0.15 – 2.11 | **0.025** |
| DOI 7 at shock | 1.15 | -0.21 – 2.50 | 0.096 |
| N _USUBJID_ | 90 | | |
| Observations | 255 | | |
